# Association of physical activity domains and television time with mental health profiles in Brazilian adolescents: a latent class analysis

**DOI:** 10.1590/0102-311XEN181324

**Published:** 2026-07-24

**Authors:** Gillianne Crystina dos Anjos Cardoso, Rafael Martins da Costa, Bruno Gonçalves Galdino da Costa, Paulo Henrique de Araújo Guerra, Jorge Mota, Roseanne Gomes Autran

**Affiliations:** 1 Universidade Federal do Amazonas, Manaus, Brasil.; 2 Universidade Federal de Rondônia, Porto Velho, Brasil.; 3 McGill University, Montreal, Canada.; 4 Universidade Estadual Paulista, São Paulo, Brasil.; 5 Faculdade de Desporto, Universidade do Porto, Porto, Portugal.

**Keywords:** Adolescent Health, Mental Health, Sedentary Lifestyle, Physical Activity, Saúde do Adolescente, Saúde Mental, Estilo de Vida Sedentário, Atividade Física, Salud del Adolescente, Salud Mental, Estilo de Vida Sedentario, Actividad Física

## Abstract

This cross-sectional study identified mental health profiles among Brazilian adolescents and examined their associations with domain-specific physical activity (leisure time, school, and active commuting) and television (TV) viewing time. Data were obtained from 144,046 adolescents in the 2019 *Brazilian National Survey of School Health* (PeNSE, acronym in Portuguese). Self-reported indicators included the frequency of five emotional states, weekly minutes of physical activity in leisure time, physical education, and commuting, and daily TV hours. Latent class analysis classified adolescents based on their mental health profiles, and multinomial logistic regression estimated associations between physical activity domains and TV viewing time with mental health classes, adjusting for covariates. Six mental health profiles were identified: (1) school-related concern; (2) anxious and stressed; (3) subthreshold internalizing; (4) anxious, sad, and angry; (5) low symptom; (6) severe internalizing. Adolescents who engaged in physical activity in their leisure time and at school were more likely to belong to the low symptom class, while those who engaged in physical activity during commuting were more likely to be in class 6 (severe internalizing). Adolescents who watched two to four hours of TV per day were more likely to belong to class 1 (school-related concern), class 4 (anxious, sad, and angry), and class 5 (low symptom), whereas those who watched TV more than four hours/day were less likely to belong to class 3 (subthreshold internalizing) and class 6 (severe internalizing). These findings underscore that both the context and quantity of physical activity and TV time are differentially related to adolescent mental health profiles, implying the need for targeted surveillance and interventions.

## Resumo

Este estudo transversal identificou perfis de saúde mental entre adolescentes brasileiros e examinou suas associações com a atividade física em domínios específicos (lazer, escola e deslocamento ativo) e com o tempo de exposição à televisão (TV). Os dados são provenientes de 144.046 adolescentes participantes da *Pesquisa Nacional de Saúde do Escolar* (PeNSE) de 2019. Os indicadores autorrelatados incluíram a frequência de cinco estados emocionais, minutos semanais de atividade física no lazer, na educação física e no deslocamento, além das horas diárias de TV. A análise de classes latentes classificou os adolescentes com base em seus perfis de saúde mental, e a regressão logística multinomial estimou as associações entre os domínios de atividade física e o tempo de TV com as classes de saúde mental, ajustando para covariáveis. Foram identificados seis perfis de saúde mental: (1) preocupação relacionada à escola; (2) ansiedade e estresse; (3) sintomas internalizantes subclínicos; (4) ansiedade, tristeza e irritação; (5) baixa sintomatologia; e (6) sintomas internalizantes graves. Os adolescentes que praticavam atividade física no lazer e na escola apresentaram maior probabilidade de pertencer à classe de baixa sintomatologia, enquanto aqueles que praticavam atividade física durante o deslocamento tiveram maior probabilidade de pertencer à classe 6 (sintomas internalizantes graves). Adolescentes que assistiam de duas a quatro horas de TV por dia apresentaram maior probabilidade de pertencer às classes 1 (preocupação relacionada à escola), 4 (ansiedade, tristeza e irritação) e 5 (baixa sintomatologia), enquanto aqueles que assistiam à TV por mais de quatro horas/dia apresentaram menor probabilidade de pertencer às classes 3 (sintomas internalizantes subclínicos) e 6 (sintomas internalizantes graves). Esses achados ressaltam que tanto o contexto quanto a quantidade de atividade física e o tempo de TV estão diferencialmente associados aos perfis de saúde mental dos adolescentes, evidenciando a necessidade de vigilância e de intervenções direcionadas.

## Resumen

Este estudio transversal identificó los perfiles de salud mental entre los adolescentes brasileños y examinó las asociaciones con la actividad física específica de cada ámbito (tiempo libre, escuela y desplazamientos activos) y el tiempo dedicado a ver la televisión (TV). Los datos proceden de 144,046 adolescentes de la *Encuesta Nacional de Salud del Escolar* (PeNSE, por su sigla en portugues) de 2019. Los indicadores autoinformados incluyeron la frecuencia de cinco estados emocionales, los minutos semanales de actividad física en el tiempo libre, la educación física y los desplazamientos, y las horas diarias de TV. El análisis de clases latentes clasificó a los adolescentes en función de sus perfiles de salud mental, y la regresión logística multinomial estimó las asociaciones entre los ámbitos de actividad física y el tiempo dedicado a ver la TV con las clases de salud mental, ajustando las covariables. Se identificaron seis perfiles de salud mental: (1) preocupación relacionada con la escuela; (2) ansiedad y estrés; (3) internalización subclínica; (4) ansiedad, tristeza y enfado; (5) síntomas leves; (6) internalización grave. Los adolescentes que realizaban actividad física en su tiempo libre y en la escuela eran más propensos a pertenecer a la clase de síntomas leves, mientras que los que realizaban actividad física durante los desplazamientos eran más propensos a pertenecer a la clase 6 (internalización grave). Los adolescentes que veían entre dos y cuatro horas de TV al día eran más propensos a pertenecer a la clase 1 (preocupación relacionada con la escuela), la clase 4 (ansiedad, tristeza e ira) y la clase 5 (síntomas leves), mientras que los que veían más de cuatro horas de TV al día eran menos propensos a pertenecer a la clase 3 (internalización subclínica) y la clase 6 (internalización grave). Estos hallazgos subrayan que tanto el contexto como la cantidad de tiempo dedicado a la actividad física y a ver la TV están relacionados de manera diferente con los perfiles de salud mental de los adolescentes, lo que implica la necesidad de una vigilancia e intervenciones específicas.

## Introduction

The mental health of adolescents is a matter of growing concern worldwide ^1^. Recent estimates indicate that approximately one in seven adolescents meets the criteria for a mental health disorder, with internalizing symptoms and suicidality representing important contributors to morbidity worldwide ^2^. Adolescence is a developmental window marked by rapid biological, physical, psychological, and social changes that increase vulnerability to emotional disorders, and symptom presentation in this period is heterogeneous ^3^ ^,^ ^4^. Cohort data from Brazil similarly suggest high and diverse burdens of anxiety and depression in youth, underscoring the need for population-sensitive approaches to identification and prevention ^3^.

Within this context, modifiable behaviors have been increasingly recognized as potential targets for mental health promotion and prevention. Physical activity and sedentary behavior are associated with mental health outcomes in adolescents ^5^ ^,^ ^6^. More specifically, evidence suggests that associations between physical activity and mental health may vary by context, including the school environment ^7^, while consistent gender differences are observed, with adolescent females reporting poorer mental health and lower levels of physical activity than males ^8^. Beyond population-level differences, emerging evidence indicates that the relationship between physical activity and mental health is domain-specific, with associations being more consistent and favorable for leisure-time physical activity, whereas other domains show weaker or more heterogeneous patterns ^9^. 

In contrast, sedentary behavior (e.g., typically measured by screen-based activities) exhibits a complex relationship with adolescent mental health ^10^ ^,^ ^11^ ^,^ ^12^. Previous studies suggest that higher levels of television viewing are associated with a greater prevalence of depressive symptoms among adolescents ^13^ ^,^ ^14^. Mechanistically, excessive passive screen time may displace restorative sleep and physical activity, contributing to behavioral profiles associated with poorer mental health ^15^. Understanding the role of screen-based sedentary behaviors in relation to mental health patterns is essential, given that adolescents spend a substantial portion of their day watching TV.

Person-centered methods such as latent class analysis (LCA) have emerged as an effective approach to capture heterogeneity in adolescent mental health by identifying homogeneous subgroups defined by symptom patterns rather than single-item thresholds ^16^. International LCA studies commonly identify three to six classes, ranging from low-symptom to high-symptom or self-harm dominant classes, and document that sociodemographic and behavioral exposures differ markedly across classes ^17^ ^,^ ^18^. Importantly, some LCA studies reveal that classes characterized by higher internalizing or comorbid symptoms tend to report less leisure time physical activity and greater screen time, whereas classes with favorable mental health profiles show higher engagement in protective behaviors ^17^ ^,^ ^18^. Such evidence suggests that mental health subgroups may differ not only in symptom severity but also in behavioral correlates that are amenable to public health action.

Despite growing evidence, important gaps persist, as most person-centered studies on behavior and mental health have been conducted in high-income settings ^19^ ^,^ ^20^. Evidence from low- and middle-income countries such as Brazil, especially from large, nationally representative samples, is scarce. Brazil’s regional, socioeconomic, and safety inequalities shape opportunities for physical activity, school physical education quality, active commuting, and screen-time patterns ^20^. Moreover, examining mental health patterns rather than isolated behaviors enables the identification of subgroups with distinct symptom profiles, supporting more targeted interventions ^3^ ^,^ ^5^. Thus, using data from a nationally representative sample of adolescents, the objectives of the current study were to: (i) identify latent mental health profiles among Brazilian adolescents; and (ii) examine how domain-specific physical activity (leisure time, school, and commuting) and TV viewing time are associated with those mental health profiles.

## Methods

### Study design

This was a cross-sectional study based on secondary data from the *Brazilian National Survey of School Health* (PeNSE, acronym in Portuguese). PeNSE 2019 is a survey conducted with the aim of monitoring risk and protective factors for the health of schoolchildren in all regions of Brazil. The research was conducted by the Brazilian Institute of Geography and Statistics (IBGE, acronym in Portuguese), with the support of the Brazilian Ministry of Health. Data from the fourth edition of PeNSE were collected from April to September 2019 via a mobile device on which the questionnaires were administered, including several health topics ^21^. For the current study, we used information related to the topics of mental health and physical activity (physical activity by domains and time spent watching TV) ^22^. 

### Ethical considerations

PeNSE 2019 was submitted to the Brazilian National Research Ethics Committee (CONEP, acronym in Portuguese) and the Brazilian National Health Council (CNS, acronym in Portuguese) and approved according to Opinion n. 3.249.268, issued on April 8, 2019 ^21^. 

### Participants and sample

Participants were selected via a geographic stratification that adopted schools from 26 capitals and the Federal District, as well as 26 other cities, totaling 53 geographic strata. The sample was defined in two stages: the first corresponding to the selection of schools, and the second to the selection of classes, which was based on the size of the school and focused on the target population of the research, students aged 13 to 17 from public and private schools. All students present on the day of the collection were invited to answer the research questionnaire ^21^.

PeNSE 2019 included 165,838 students enrolled in the 7th to 9th grades of elementary school and the 1st to 3rd grades of high school. However, complete information on the variables studied was only available for 144,046 students, representing 86.9% of the total sample ^21^ ^,^ ^22^.

### Variables

#### Mental health

Mental health was measured through five questions that reflected the adolescents’ concerns and feelings in the previous thirty days, as follows: (1) “How often did you feel very worried about common things in your daily life, such as school activities, sports competitions, homework, etc.?”; (2) “How often did you feel sad?”; (3) “How often did you feel like no one cared about you?”; (4) “How often did you feel irritable, nervous, or in a bad mood because of something?”; and (5) “How often did you feel that life is not worth living?”, with answer options: never; rarely; sometimes; mostly; always. These answers were classified into three categories: low frequency, sometimes, and high frequency.

#### Physical activity

Three domains of physical activity (leisure time, school, and commuting) were assessed using previously validated questions ^23^. The choice of these domains is based on the following: the leisure-time physical activity domain constitutes a context with high protective potential for adolescents’ mental health, as it is consistently associated with enhanced self-esteem, strengthened social relationships, and a reduction in internalizing symptoms ^24^. 

School-based physical activity is a key domain for adolescent physical activity and development, offering unparalleled opportunities for socialization and the adoption of healthy behaviors ^25^. Within this setting, physical education classes, especially when regular or enhanced with targeted interventions, have been shown to increase participation in overall physical activity, improve socioemotional skills, and reduce anxiety and depressive symptoms ^7^ ^,^ ^26^. 

Evidence from adolescents across various countries suggests that active commuting may contribute to lower levels of depressive symptoms and greater overall well-being ^27^ ^,^ ^28^. In addition to contributing to overall physical activity, it promotes autonomy, daily routine structure, and social interaction with peers, and all these aspects are deeply related to mental health and well-being ^28^. 

To evaluate physical activity across each domain selected for this study, the adolescents responded to the following questions: (1) “In the last seven days, excluding physical education classes at school, how many days did you practice any physical activity, including sports, dance, gymnastics, weight training, wrestling, or other activities (response range 0-7)?”; (2) “In addition to physical education classes, how much time each day did you participate in these activities (such as sports, dancing, gymnastics, resistance training, wrestling, or other activities) (response range from less than 10 minutes to 1 hour or more)?”. Leisure time physical activity was obtained by multiplying the weekly frequency by the daily duration. The recommendation for moderate-to-vigorous physical activity in adolescents is, on average, 60 minutes/day (or 420 minutes/week) ^29^.

Considering only school days (i.e., five days), leisure-time physical activity was categorized as does not engage in (60 minutes × 0 days), ≤ 299 minutes/week (60 minutes × 1-4 days), and ≥ 300 minutes/week (60 minutes × 5 or more days).

The volume of physical activity in physical education classes was assessed through two questions: (1) “In the last seven days, how many days did you have physical education classes at school (response range 0-5)?”; (2) “In the last seven days, how much time per day did you practice physical activity or sports during physical education classes at school (response ranges from less than 10 minutes to 1 hour or more)?”. The volume of physical education classes was obtained by multiplying the weekly frequency by the daily duration of these physical activity. It was categorized as does not engage in, ≤ 45 minutes/week (one physical education class per week), and ≥ 46 minutes/week (two or more physical education classes per week) ^29^.

In total, four questions were used to measure commuting to and from school: (1) “How many days did you walk or bike to/from school in the past seven days?”; (2) “When you walk or bike to school, how much time do you spend walking or biking to school?”. Responses to frequency questions ranged from none to 7 days, and duration questions ranged from less than 10 minutes to 1 hour or more per day. The volume of active travel to and from school (calculated separately) was obtained by multiplying the frequency to/from school. Thus, the total volume of physical activity during commuting was categorized as does not perform active commuting, 1-149 minutes/week, and ≥ 150 minutes/week ^30^.

#### Television viewing time

Evidence from systematic reviews ^6^ ^,^ ^31^ and large-scale studies ^11^ ^,^ ^32^ links excessive TV viewing with greater internalizing symptoms, poorer well-being, and higher risks of depression, anxiety, and behavioral problems.

TV viewing time was measured by means of a question that did not consider Saturdays, Sundays, or holidays: (1) “How many hours a day do you watch TV?”. Adolescents reported the number of hours spent watching television per day, ranging from no TV viewing to more than 8 hours per day. TV viewing time was categorized as < 2 hours/day, 2-4 hours/day, and > 4 hours/day ^33^.

### Covariates

The variables considered for analysis were sex (male and female), age group, maternal education, and living with parents. Age was categorized into three groups: ≤ 12 years, 13-17 years, and ≥ 18 years. Maternal education was classified as ≤ 8 years (did not study, incomplete elementary school, and complete elementary school), 9 to 12 years (incomplete high school and complete high school), ≥ 13 years (incomplete higher education and complete higher education), and don’t know. Living with parents was categorized as both (lives with mother and father), only with mother, only with father, and neither of them.

### Statistical analysis

Participant characteristics are described using absolute and relative frequencies. Latent class analysis was applied to the five categorical mental health indicators listed above to identify latent symptom classes. LCA is one of the finite mixed models, a multivariate analysis method that aims to identify the number of behavioral profiles considering mental health indicators in the present study. A range of latent class models was computed to identify the suitable number of classes that best represent the subgroups of mental health problems in this sample. This started with a two-class model and progressively included models with an increasing number of classes ^34^. Then, statistical model adjustment indices, such as maximum log-likelihood, Akaike information criteria (AIC), Bayesian information criteria (BIC), sample-size adjusted BIC (SABIC), and consistent AIC (CAIC), were used to identify the final number of classes ^34^. LCA was performed using the poLCA package version 1.6.0.1 (https://www.rdocumentation.org/packages/poLCA/versions/1.6.0.2), without sampling weights. Because the poLCA package does not fully support design-based estimation for clustered complex surveys, sampling-design effects were incorporated in the association analysis rather than in model fitting: class membership (modal assignment) and posterior probabilities were exported. Multinomial logistic regression models, used to verify the associations of physical activity domains and TV time with mental health classes, were fitted using the survey package version 4.2-1, with the full PeNSE complex-sample specification (i.e., strata, clusters, and sample weights) to obtain design-corrected odds ratios (OR) and 95% confidence intervals (95%CI). All analyses were performed in the statistical language R 4.3.2 (http://www.r-project.org). A significance level of 5% was assumed.

## Results

Table 1 shows the characteristics of the participants. Regarding the mental health characteristics of the sample under investigation, slightly over half of the participants reported experiencing a high frequency of concerns related to school activities. Conversely, the most prevalent responses to feelings of sadness, perceived lack of care, and a sense of life’s insignificance were indicative of low frequency. 

**Table 1:** Description of the sample of Brazilian schoolchildren. *Brazilian National Survey of School Health* (PeNSE), 2019.

Variables	Total (144,046)	
	n	%
Concern about school activities		
Low frequency	26,074	21.6
Sometimes	38,187	28.0
High frequency	79,785	50.4
Sadness		
Low frequency	48,590	34.6
Sometimes	51,242	34.5
High frequency	44,214	30.9
Feeling like no one cares about you		
Low frequency	67,892	46.4
Sometimes	35,252	24.1
High frequency	40,902	29.5
Nervousness, anger, bad mood		
Low frequency	36,403	26.7
Sometimes	48,986	32.8
High frequency	58,657	40.5
Feeling that life is not worth living		
Low frequency	92,566	62.9
Sometimes	23,038	16.2
High frequency	28,442	20.9
Leisure time physical activity (minutes/week)		
Does not engage in	70,869	49.2
≤ 299	63,043	44.2
≥ 300	10,134	6.6
School physical activity (minutes/week)		
Does not engage in	62,528	44.9
≤ 45	39,765	27.2
≥ 46	41,753	27.9
Commuting physical activity (minutes/week)		
Does not engage in	68,798	38.5
≤ 149	60,655	48.7
≥ 150	14,593	12.8
Television time (hours/day)		
< 2	95,481	63.7
2-4	26,500	18.8
> 4	22,065	17.5
Sex		
Male	68,331	47.2
Female	75,715	52.8
Age group (years)		
≤ 12	22,685	12.7
13-17	113,718	80.4
≥ 18	7,643	6.9
Maternal education (years)		
≤ 8	21,245	22.7
9-12	42,249	33.0
≥ 13	55,782	23.0
Not known	24,770	21.3
Lives with parents		
Both	84,392	55.5
Only with mother	43,605	32.2
Only with father	6,593	4.8
Neither	9,456	7.5

Table 2 presents information on the assessment of the most parsimonious quantity of latent class models of mental health indicators in Brazilian schoolchildren. The six-class model was selected as the best-fitting model as it presented the lowest values of all the indicators included.

**Table 2:** Evaluation information of the best model for latent class formation. *Brazilian National Survey of School Health* (PeNSE), 2019.

Classes	Maximum log-likelihood	BIC	SABIC	AIC	CAIC
2	-743311.6	1486876	1486809	1486665	1486897
3	-725880.5	1452146	1452044	1451825	1452178
4	-722016.7	1444550	1444414	1444119	1444593
5	-721640.5	1443930	1443758	1443389	1443984
6	-720417.6	1441616	1441410	1440965	1441681

AIC: Akaike information criteria; BIC: Bayesian information criteria; CAIC: consistent AIC; SABIC: sample-adjusted BIC.

Regarding latent class formation (Table 3), item-response probabilities (conditional probabilities of each response category given class membership) indicate the modal response for each mental health indicator within each class. Class 1 was denoted as school-related concern (20.06%): the highest probabilities were observed for high frequency concern about school activities (p = 0.578), sometimes feeling sad (p = 0.776), low frequency of feeling that no one cares (p = 0.556), sometimes feeling nervousness/angry/bad mood (p = 0.597), and low frequency feeling that life is not worth living (p = 0.844). 

**Table 3:** Prevalence and item-response probabilities of each mental health indicator for each class in Brazilian schoolchildren (n = 144,046). *Brazilian National Survey of School Health* (PeNSE), 2019.

Mental health indicators/Categories	Class 1 (20.06%)	Class 2 (9.00%)	Class 3 (4.82%)	Class 4 (13.52%)	Class 5 (34.17%)	Class 6 (17.83%)
Concern about school activities						
Low frequency	0.090	0.148	0.403	0.045	0.325	0.112
Sometimes	0.332	0.195	0.302	0.203	0.346	0.133
High frequency	0.578	0.657	0.294	0.752	0.329	0.755
Sadness						
Low frequency	0.185	0.329	0.241	0.006	0.867	0.005
Sometimes	0.776	0.493	0.368	0.449	0.128	0.029
High frequency	0.039	0.178	0.391	0.545	0.005	0.967
Feeling like no one cares about you						
Low frequency	0.556	0.593	0.233	0.093	0.879	0.051
Sometimes	0.378	0.246	0.314	0.525	0.074	0.081
High frequency	0.066	0.161	0.453	0.383	0.047	0.868
Nervousness, anger, bad mood						
Low frequency	0.128	0.248	0.317	0.081	0.556	0.042
Sometimes	0.597	0.191	0.282	0.33	0.365	0.133
High frequency	0.275	0.561	0.401	0.589	0.079	0.825
Feeling that life is not worth living						
Low frequency	0.844	0.897	0.249	0.408	0.958	0.072
Sometimes	0.141	0.053	0.34	0.436	0.026	0.159
High frequency	0.016	0.05	0.411	0.156	0.016	0.769

Note: class 1: school-related concern; class 2: anxious and stressed; class 3: subthreshold internalizing class 4: anxious, sad, and angry; class 5: low symptom; class 6: severe internalizing.

Class 2 was denoted as anxious and stressed (9.00%): modal responses included high frequency concern about school (p = 0.657), sometimes sadness (p = 0.493), low frequency feeling no one cares (p = 0.593), high frequency of nervousness/anger/bad mood (p = 0.561), and low frequency of feeling that life is not worth living (p = 0.897). Class 3 was denoted as subthreshold internalizing (4.82%): modal responses were low frequency concern about school (p = 0.403) and high frequency for sadness (p = 0.391), sometimes feeling no one cares (p = 0.453), nervousness/angry/bad mood (p = 0.401), and feeling that life is not worth living (p = 0.411). Class 4 was denoted as anxious, sad, and angry (13.52%): characterized by high frequency of concern about school (p = 0.752), high frequency of nervousness/angry/bad mood (p = 0.589), and sometimes for the feeling that life is not worth living (p = 0.436). 

Class 5 was denoted as low symptom (34.17%): predominantly low frequency responses across indicators (sadness, p = 0.867; feeling that no one cares, p = 0.879; nervousness/angry/bad mood, p = 0.556; feeling that life is not worth living, p = 0.958), with sometimes being modal only for concern about school activities (p = 0.346). Finally, class 6 was denoted as severe internalizing (17.83%): uniformly high probabilities of endorsing high frequency for all indicators (concern about school, p = 0.755; sadness, p = 0.967; feeling that no one cares, p = 0.868; nervousness/angry/bad mood, p = 0.825; feeling that life is not worth living, p = 0.769). These item-response probabilities indicate that classes 3, 4, and 6 reflect elevated endorsement of internalizing symptoms, whereas class 5 denotes a favorable profile.

Table 4 presents associations between physical activity domains, TV viewing, and latent mental health classes. Class 6, showing the poorest mental health, was set as the reference in the multinomial logistic regression; OR > 1.0 indicate higher odds of belonging to the target class than to class 6 for the exposed group.

**Table 4:** Associations between the domains of physical activity, television time, and sociodemographic aspects with six latent class of mental health indicators in Brazilian schoolchildren (n = 144,046). *Brazilian National Survey of School Health* (PeNSE), 2019.

Parameter	Class 6 vs. class 1	Class 6 vs. class 2	Class 6 vs. class 3	Class 6 vs. class 4	Class 6 vs. class 5
OR (95%CI)	OR (95%CI)	OR (95%CI)	OR (95%CI)	OR (95%CI)
Leisure-time physical activity (minutes/week)					
Does not engage in	Reference	Reference	Reference	Reference	Reference
≤ 299	1.31 (1.20; 1.43)	1.15 (1.03; 1.28)	1.47 (1.30; 1.66)	1.30 (1.18; 1.43)	1.51 (1.39; 1.64)
≥ 300	1.09 (0.92; 1.30)	1.18 (0.95; 1.47)	1.26 (0.96; 1.64)	1.18 (0.97; 1.44)	1.45 (1.23; 1.71)
Physical education classes (minutes/week)					
Does not engage in	Reference	Reference	Reference	Reference	Reference
≤ 45	1.14 (1.04; 1.26)	1.09 (0.96; 1.24)	1.05 (0.92; 1.21)	1.04 (0.94; 1.16)	1.10 (1.01; 1.21)
≥ 46	1.13 (1.02; 1.25)	1.13 (0.99; 1.28)	0.87 (0.75; 1.01)	0.98 (0.88; 1.09)	1.19 (1.08; 1.31)
Active commuting (minutes/week)					
Does not engage in	Reference	Reference	Reference	Reference	Reference
≤ 149	0.83 (0.77; 0.90)	0.83 (0.76; 0.92)	0.98 (0.87; 1.10)	0.96 (0.88; 1.05)	0.84 (0.80; 0.93)
≥ 150	0.56 (0.50; 0.63)	0.61 (0.52; 0.70)	0.86 (0.74; 1.02)	0.76 (0.67; 0.86)	0.50 (0.45; 0.56)
Television time (hours/day)					
< 2	Reference	Reference	Reference	Reference	Reference
2-4	1.17 (1.06; 1.29)	1.04 (0.92; 1.18)	1.13 (0.98; 1.30)	1.13 (1.02; 1.26)	1.13 (1.02; 1.24)
> 4	0.76 (0.69; 0.83)	0.86 (0.79; 1.00)	1.16 (1.02; 1.33)	0.81 (0.73; 0.90)	0.75 (0.68; 0.82)

95%CI: 95% confidence interval; class 1: school-related concern; class 2: anxious and stressed; class 3: subthreshold internalizing class 4: anxious, sad, and angry; class 5: low symptom; class 6: severe internalizing; OR: odds ratio.

Note: model adjusted for sex, age group, maternal education, and living with parents. Bold values represent p < 0.05.

For leisure-time physical activity, those reporting ≤ 299 minutes/week had higher odds of membership in all less severe classes relative to class 6. In contrast, adolescents reporting ≥ 300 minutes/week of leisure physical activity showed no associations for most classes, with the notable exception of class 5 (Table 4). These patterns suggest that any leisure physical activity (versus none) was associated with greater odds of belonging to less severe profiles rather than class 6.

For school-based physical activity (physical education classes), compared with inactive participants, both categories (≤ 45 minutes/week and ≥ 46 minutes/week) were associated with modestly increased odds of membership in class 1 and class 5 relative to class 6. These effect sizes were small but consistent with a positive association between school physical activity (physical education classes) and membership in classes with less severe symptoms (Table 4).

Conversely, active commuting showed an inverse pattern. Adolescents who engaged in active commuting had substantially lower odds of being in the less severe classes (e.g., class 1, class 2, class 4, and class 5) relative to class 6 (Table 4). TV time exhibited a mixed pattern across classes. Moderate TV time (2-4 hours/day) and high TV time (> 4 hours/day) were differentially associated with class membership. For instance, for some comparisons (e.g., class 3), higher TV time was associated with greater odds vs. class 6, whereas for others (e.g., class 5), higher TV time was associated with lower odds vs. class 6 (Table 4).

Overall, when using class 6 as the reference, the pattern of associations suggests that leisure and school (physical activity classes) physical education are more often associated with higher odds of being in less severe, or favorable, mental health profiles, whereas active commuting is associated with lower odds of being in less severe classes relative to the severe profile. TV time associations were mixed and require cautious interpretation.

## Discussion

The current study aimed to identify distinct profiles of Brazilian adolescents based on mental health indicators and to examine the association of physical activity by domains and TV time with these profiles. A total of six distinct mental health profiles were identified. Class 5 represented a relatively resilient subgroup with lower probabilities of sadness, suicidal thoughts, and irritability/anger. In contrast, class 6 included adolescents at higher risk, showing elevated probabilities across all negative symptoms. Classes 1 and 2 were dominated by anxiety and stress, often linked to school pressures, while classes 3 and 4 exhibited intermediate emotional distress, including sadness, irritability, and feelings of worthlessness.

This pattern aligns with other LCA studies, which often identify a predominant subgroup with lower probabilities of internalizing symptoms and smaller subgroups with progressively higher probabilities of such symptoms ^35^ ^,^ ^36^. However, symptom-focused LCA indicators do not equate Low symptom probabilities with positive mental health (e.g., flourishing), which encompasses emotional, psychological, and social well-being. The two-continua model distinguishes mental illness from positive mental health, so the absence of symptoms does not imply well-being ^37^. For instance, Piqueras et al. ^36^ identified four latent profiles of youth emotional symptoms, while Ling et al. ^35^ reported low-, middle-, and high-risk classes in Chinese adolescents.

Recognition of these subgroups is epidemiologically relevant and informs interventions; while one-third of adolescents are symptom-free, those with severe internalizing symptoms may require clinical care, and those with academic anxiety may benefit from school-based stress reduction. In short, this person-centered typology aids surveillance and points to tailored prevention and/or intervention strategies ^35^ ^,^ ^36^.

Moreover, the consistent association between school-related concerns and other emotional symptoms, such as sadness, nervousness, anger, and feelings of worthlessness, and internalizing symptoms underscores the role of the school environment as a determinant of adolescent mental health. These findings suggest that academic demands and the school environment not only act as specific stressors but also interact with broader dimensions of mental health. In a longitudinal study conducted with adolescents, results showed that school environments are predictive of depressive symptoms ^38^. 

Thus, schools are strategic settings for early identification of psychological distress and for interventions that promote healthier environments, reduce pressures, and enhance social-emotional support. Our findings underscore the heterogeneity of adolescent mental health profiles, consistent with international studies ^39^ showing both resilient and high-risk subgroups. In Brazil, this diversity reflects socioeconomic and regional inequalities, which also shape opportunities for engaging in various types of physical activity ^40^.

In light of the associations across different physical activity domains, leisure-time physical activity showed a counterintuitive dose pattern. Adolescents who reported performing leisure physical activity (≤ 299 minutes/week) had consistently higher odds of belonging to all less severe classes relative to class 6, whereas meeting or exceeding ≥ 300 minutes/week did not yield additional consistent associations except for class 5. This pattern suggests that, at the population level, the primary mental health gain may accrue in the transition from inactivity to some leisure physical activity rather than from moderate to high volumes. From a public health perspective in Brazil, where barriers to sustained high-volume physical activity exist, promoting the initiation and maintenance of any leisure physical activity may be a pragmatic and high-yield strategy ^41^.

Increased physical activity in leisure time supports better mental health and reduces stress symptoms compared with inactive adolescents. This finding reinforces the priority of promoting leisure time physical activity within a broader perspective related to human development, which involves − in addition to energy expenditure − issues such as meanings, pleasure, self-esteem, and strengthening social support networks, which serve as protective factors against symptoms of anxiety and depression ^42^. Although our findings reinforce the positive association between leisure-time physical activity and a lower frequency of negative indicators of mental health, the prevalence of physical inactivity among Brazilian adolescents remains high ^43^. It is important to note that access to a safe space for physical activity during leisure time may be limited in the Brazilian context, particularly in socioeconomically vulnerable communities. Thus, ensuring adolescents have access to secure and enjoyable spaces for leisure-time physical activity is critical for promoting sustained engagement and enhancing overall well-being ^44^.

The findings also indicate that participation in school-based physical education was associated with less severe mental health profiles, with adolescents showing a higher likelihood of belonging to the school-related concern and low symptom classes. It is worth highlighting that the Brazilian National Curriculum Base (BNCC, acronym in Portuguese) for physical education in basic education indicates that school classes should provide different experiences related to movement, allowing students to participate autonomously in leisure and health contexts, without an instrumental perspective. Moreover, other studies suggest that regular participation in physical education classes is linked to lower levels of anxiety and depressive symptoms, reinforcing the role of the school as a protective environment for adolescent mental health ^7^ ^,^ ^45^.

Furthermore, a review study highlights that well-structured physical education programs not only improve physical fitness but also enhance self-esteem, resilience, and social connectedness, which are key protective factors against internalizing problems ^9^. International studies emphasize that the psychosocial benefits of physical education are maximized when classes adopt inclusive and supportive pedagogical approaches, providing opportunities for all adolescents to engage meaningfully in physical activity ^46^.

Regarding active commuting, our results indicate that adolescents who actively commute to school were more likely to belong to class 6 compared with most other mental health classes, contrasting with patterns for leisure-time and school physical activity. Although active commuting is linked to positive health indicators ^47^ ^,^ ^48^ and can reduce stress levels ^27^ ^,^ ^49^, in Brazil it is often involuntary due to socioeconomic vulnerabilities ^40^ ^,^ ^50^, and environmental challenges such as insecurity, urban violence, long distances, traffic, and poor infrastructure ^51^. Consequently, active commuting may reflect social inequities and confer mental health benefits only when supported by safe infrastructure, reduced violence, and integrated mobility policies.

When examining the association between TV time and mental health, the results obtained from the study sample indicate mixed patterns. Adolescents who watched two to four hours of TV per day were more likely to be in class 1, class 4, and class 5. Even moderate levels of TV exposure have been associated with school-related concerns and emotional difficulties among adolescents. The literature indicates that regular TV viewing can impair sleep quality, reduce study time and attention, and increase social comparisons and body dissatisfaction ^13^ ^,^ ^32^. 

Adolescents who watched more than four hours of TV per day were more likely to be in class 3 and class 6. Studies have shown that high screen time is associated with poor mental health conditions, such as anxiety and depression ^13^ ^,^ ^14^. Although other types of screen time, such as computers, smartphones, and tablets, are integrated into adolescents’ daily routines, watching TV remains one of the predominant forms of sedentary leisure. Future questionnaires of PeNSE should consider the evaluation of social media and online gaming, which are increasingly relevant to Brazilian adolescents, as they interact with mental health outcomes differently from traditional TV exposure.

Given their public health and policy relevance, the identification of distinct mental health profiles supports tailored, rather than “one-size-fits-all”, interventions. Key implications include prioritizing leisure-time physical activity among inactive adolescents to achieve meaningful mental health gains, strengthening school-based physical education where access to safe leisure spaces is limited, and ensuring that active commuting is supported by safe infrastructure and mobility policies. Screen-time recommendations should be context-sensitive, balancing limits on harmful content and duration with efforts to expand alternative leisure opportunities.

The results of this study should be considered in light of some strengths, including that it used data from PeNSE, a periodic survey that enables nationally representative estimates considering schools in both urban and rural areas, as well as the uniqueness of the findings. Another important aspect was the type of analysis conducted in this research, LCA, which was able to identify mental health profiles among Brazilian adolescents, thus raising questions about the mental health of this population in Brazil.

However, some limitations should be noted. First, the self-reported questionnaire may lead to inaccurate responses by students due to recall bias. For future studies, we recommend some improvements, such as the validation of the instrument that measures the adolescent mental health, and more information about the type of physical activity that these adolescents were exposed to, as well as the measurement of other components of screen time (smartphones, computers, video games, etc.). Moreover, investigating only regularly enrolled students present on the day of the survey excludes adolescents who are not part of the education system, who may be more vulnerable, and therefore does not capture all adolescents in their diverse realities. 

## Conclusion

Brazilian adolescents are heterogeneous in mental health symptoms, with distinct subgroups differing in behavioral correlates. Leisure and school physical activity are more strongly associated with less severe profiles, with the largest marginal gains observed when adolescents move from inactivity to any leisure physical activity. Active commuting and TV time show context-dependent relationships that reflect broader social and environmental determinants. Policies that promote the initiation of leisure physical activity, strengthen inclusive and quality physical education, and integrate urban safety and mobility investments are likely to produce the most equitable mental health benefits for adolescents in Brazil.

## Data Availability

The sources of information used in the study are indicated in the body of the article.
